# Genomic and phylogenetic analysis of choriolysins, and biological activity of hatching liquid in the flatfish Senegalese sole

**DOI:** 10.1371/journal.pone.0225666

**Published:** 2019-12-05

**Authors:** Carlos Carballo, Evangelia G. Chronopoulou, Sophia Letsiou, Eleni Spanidi, Konstantinos Gardikis, Nikolaos E. Labrou, Manuel Manchado

**Affiliations:** 1 IFAPA Centro El Toruño, Junta de Andalucía, El Puerto de Santa María, Spain; 2 Laboratory of Enzyme Technology, Department of Biotechnology, School of Applied Biology and Biotechnology, Agricultural University of Athens, Athens, Greece; 3 Research and Development Department, APIVITA S.A., Athens, Greece; Laboratoire de Biologie du Développement de Villefranche-sur-Mer, FRANCE

## Abstract

The hatching enzymes or choriolysins are key proteases in fish life cycle controlling the release of larvae to surrounding environment that have been suggested as target for novel biotechnological uses. Due to the large amounts of eggs released by the flatfish *Solea senegalensis*, during the spawning season, the hatching liquid properties and choriolysin-encoding genes were investigated in this species. A genomic analysis identified four putative genes referred to as SseHCEa, SseHCEb, SseLCE and SseHE. The phylogenetic analysis classified these paralogs into two clades, the clade I containing SseHCE paralogs and the clade II containing two well-supported subclades named as HE and LCE. The two SseHCE paralogs were intron-less and both genes were tandemly arrayed very close in the genome. The synteny and gene rearrangement identified in the flatfish lineage indicated that the duplication of these two paralogs occurred recently and they are under divergent evolution. The genes SseHE and SseLCE were structured in 8 exons and 7 introns and the synteny was conserved in teleosts. Expression studies confirmed that the four genes were expressed in the hatching gland cells and they migrate co-ordinately from the head to around the yolk sac close to the hatch with specific temporal and intensity expression profiles. Although the mRNA levels of the four genes peaked in the hours previous to larval hatching, the SseHCE and SseLCE paralogs kept a longer expression than SseHE after hatching. These expression patterns were consistent even when larvae were incubated at different temperatures that modified hatching times. The analysis of hatching-liquid using SDS-PAGE and zymography analyses of hatching liquid identified a major band of expected choriolysin size. The optimal pH for protease activity was 8.5 and inhibition assays using EDTA demonstrated that most of the activity in the hatching liquid was due to metalloproteases with Ca^2+^ ions acting as the most effective metal to restore the activity. All these data provide new clues about the choriolysin evolution and function in flatfish with impact in the aquaculture and the blue cosmetic industry.

## Introduction

The hatching enzymes, also known as choriolysins, are essential proteases for reproduction success and larval survival. They facilitate egg envelope digestion to release the larva from the chorion to the surrounding environment. Structurally, these proteases are highly evolutionary conserved in vertebrates and they belong to the astacin family of zinc-dependent metalloproteases [1–3]. According to their gene structure and evolution, they are classified into two main classes named as high choriolytic (HCE) and low choriolytic (LCE) enzymes [4, 5]. As the embryo develops, these enzymes accumulate in the hatching gland cells as proenzymes and they are secreted and activated at the onset of larval hatching. Both enzyme classes act cooperatively to disintegrate the egg envelope in a stepwise manner with HCE swelling the chorion and LCE fully solubilizing of chorionic proteins [4, 6]. Although these two choriolysin classes are conserved in most teleosts, the number of genes encoding them is highly variable across species due to the teleost-specific genome duplication (3R) and retrocopy mechanisms [5–8]. This gene redundancy has resulted in the sub-functionalization of some paralogs [6] or neo-functionalization with the recognition of new cleavage sites [4] or regulation of ovary atresia [9]. Due to the importance of these proteases in fish life cycle and the different evolution in lineages, the set of choriolytic enzymes needs to be characterized in a species-specific way. This is of particular interest in those fish produced in aquaculture such as the Senegalese sole (*Solea senegalensis*) in which the enzymes play a key role in the quality and survival of hatched larvae and an excess volume of fertilized eggs are produced during the spawning season that can be used for biotechnological purposes.

The distribution and amounts of hatching gland cells in the chorion determine the pattern of envelope digestion [10, 11]. In the Pleuronectiformes spotted halibut (*Verasper variegatus*) and Atlantic halibut (*Hippoglossus hippoglossus*), the hatching gland cells group as a ring-shaped belt that split the envelope into two rigid parts [8, 12] while in the flatfish marbled flounder (*Pseudopleuronectes yokohamae*) the hatching gland cells spread over the yolk sac creating a big hole for the embryo to escape [8]. Moreover, the total protease levels are highly dependent on the chorion thickness and developmental times [8, 11]. A precise characterization of choriolysin expression patterns and their levels appear crucial to understand the co-evolution with egg structure and the strategies used by the embryos for hatch and optimize handling of these early fish stages.

The Senegalese sole is a valuable flatfish mainly produced in Southern Europe but with markets worldwide [13, 14]. This species spawns mainly in spring releasing high amounts of small eggs (<1 mm) that hatch in 24 hours if incubated at 20°C [15, 16]. The manipulation thermal regimes during embryonic stages to epigenetically reprogram growth performance is a new practice in hatchery procedures [16–18] but a better knowledge of mechanisms controlling hatching are required to refine and improve larval survival. Moreover, proteases, are being used in cosmetics as skin cleansing agents for moisturization and improvements in skin surface characteristics and appearance and for the reduction of cutaneous inflammation. These uses have triggered the interest of "blue cosmetic" based-industry on choriolysins as a potential natural ingredient for skin care [19] that could promote the circular economy strategy.

In this work, the genes encoding the HCE and LCE choriolysins and their genomic structures were identified by *in silico* analysis and the expression patterns during embryo development, content and the regulation by temperature were determined. Finally, the protein profile, proteolytic activity and bioactivity of hatching liquids on fibroblasts were studied demonstrating the metalloprotease nature of protease activity and the ability of some metals and chelators to modulate this activity.

## Materials and methods

### Fish samples

Two trials using eggs collected from natural spawns using a wild Senegalese sole broodstock kept at “El Toruño” facilities (El Puerto de Santa María, Cádiz) were carried out. In both trials, the fertilized eggs were separated by buoyancy in a 1,000 mL measuring cylinder and distributed in a 3L-plastic jar containing seawater (37 ppt) with gentle aeration until use in triplicate. Embryos at the onset of the trials were in gastrula stage (50% epiboly) and they were incubated at 20°C and at an initial density of 2,000 embryo L^-1^. Developmental stages were monitored on the microscope before samplings following the classification previously reported [16]. In the first trial, a total of 100 embryos were sampled in pharyngula at 6 h and 1 h before hatching, just at hatch and at 6 h and 26 h post-hatch. Larvae were sacrificed in anaesthesia overdose (MS-222; 300 mg ml^-1^), rinsed in distilled water and fixed in paraformaldehyde as previously described [20, 21], dehydrated in ethanol and stored at -20°C until use for whole-mount *in situ* hybridization (WISH). A second trial for the quantification of choriolysin mRNA levels was also carried out. Embryos were handled and monitored as indicated above and larvae were sampled in gastrula, segmentation, in pharyngula at 10h, 7h and 4h before hatching, just at hatch and 8 h and 56 h post-hatch. Embryos and larvae were sacrificed by anaesthesia overdose as indicated above, rinsed by distilled water, fixed in RNA-later (Invitrogen) and stored at -80°C until use for RNA extraction. Samples for the evaluation of the choriolysin expression in embryos incubated at different temperatures were those previously published in an unrelated study [21]. Shortly, incubation temperatures (16 and 20°C) were selected considering the thermal range in which wild broodstocks naturally spawns and larvae develop correctly. The time until hatch was highly dependent on temperature and spanned for 21h at 20°C and 53h at 16°C. To standardize the temporal framework due to the difference in development associated with the temperatures, embryonic stages were classified as follows: gastrula, segmentation and hatch.

All procedures were authorized by the Bioethics and Animal Welfare Committee of IFAPA and given the registration number 26-11-15-374 by the National authorities for the regulation of animal care and experimentation.

### Sequence analysis and phylogeny

Choriolysin sequences were identified after performing a search in the databases SoleaDBv3 and v4.1 [22]. The unigeneID of selected transcripts are indicated in S1 Table. Sequences were edited using EditSeq and aligned using MegAlign v8.3 (DNASTAR). Genome scaffolds were identified by blasting the cDNA sequencies onto a reference a draft genome assembled by Nanopore technology using BLAST^®^ v2.2.18+ for macosX. To identify the intron and exon structure, the cDNAs were aligned with target scaffolds using Seqman *v*8.2 (DNASTAR). Sequences were deposited in GenBank/EMBL/DDBJ with accession numbers MK789467- MK789473. The signal peptide cleavage site was predicted with SignalIP (http://www.cbs.dtu.dk/services/SignalP/). Known protein domains were identified using Interpro (http://www.ebi.ac.uk/interpro/) and SMART (http://smart.embl-heidelberg.de/). Prediction of the three-dimensional structure was carried out by SWISS-MODEL (http://swissmodel.expasy.org/).

To carry out the phylogenetic analysis, sequences from other fish encoding for choriolysins and astacin-like proteases were retrieved from GenBank and Ensembl (S1 Table). The Maximum-likelihood phylogenetic tree was built using iq-Tree v1.6.0 [23] under the WAG model with a 6-categories free-rate distribution and empirical codon models (WAG+F+R6) that was selected as the best-fitting model according to AIC. Statistical supports were drawn from 1000 ultrafast bootstrap values with a 0.99 minimum correlation as convergence criterion [24]. Synteny analysis was carried out using *Genomicus* genome browser (http://www.genomicus.biologie.ens.fr).

### Whole *in situ* hybridization

WISH methods were those previously optimized for sole [20, 21, 25]. Embryos before hatching were manually dechorionated using fine forceps A total of n = 10 larvae per sampling point and n = 5 specimens for negative controls were analysed. To synthetize choriolysin probes, a gene fragment of each choriolysin gene was PCR amplified using targeted gene-specific primers (Table 1 and S1 File). In the case of SseHCEa and SseHCEb, primers were located in 3'-UTR. Primers were designed using Oligo v6.89 software (Medprobe). PCR reactions for probe amplification were carried out using cDNA of a larval pool as template. PCR products were cloned in TOPO-TA vector and later sequenced using a BigDye^®^ Terminator v3.1 kit (Applied Biosystems). Sense and anti-sense probes were prepared as previously described [20, 21, 25].

**Table 1:** Primers used for qPCR and WISH analysis. Amplicon size is indicated. For probe amplification, one of the primers was the same used in the qPCR analysis.

Name	Primer	Sequence (5’→3’)	Amplicon (bp)
SseLCEq_1	F	ACTCCAACCTACGACCCGTCTGCTACCAT	102
SseLCEq_2	R	ACTGAAGCCCCAGCACCTGTAGAGCTTG	
SseLCEprobe_1	F	ACGGATGCTCCTCTTTGCTGGGTTACAC	401*
SseHEq_1	F	GTTGGCAGAGACGGGGGACATCAGGT	129
SseHEq_2	R	GTCCCTGTCGCTCCTCGTGTGCTCGT	
SseHEprobe_1	F	CTCCTCTCTGCTGTCTTCACCGTCCTGCT	540^‡^
SseHceboth_1	F	CTGTTGCTGCTCCTGCTCGGCCTC	93
SseHceboth_2	R	AATCCTGGTGGTGATGTCAACGTCGTCT	
SseHceaq_1	F	AATCATCACGACTGTTGTTGTATCAGTTTT	125
SseHceaq_2	R	CAACAGACATTCATGCAGCTTTAGCAATCC	
SseHcebq_1	F	AACAGCATGACTGCTATTGTCATTTCTAGG	118
SseHcebq_2	R	GGCCATTCTTCCTTTGCTCAGTGATTGTAT	
SseHceaprobe_1	F	ACAACTTCTACAAGCAGAACACCAACAACC	490^†^ 455^$^

*SseLCEprobe_1/SseLCEq_2

^‡^SseHEprobe_1/SseHEq_2

^†^SseHceaprobe_1/SseHceaq_2

^$^SseHceaprobe_1/SseHcebq_2

### RNA isolation and gene expression analysis

Embryo and larval samples (n = 3 pools containing 15–20 individuals) were homogenized in a Fast-prep FG120 instrument (Bio101) using Lysing Matrix D (Q-Bio-Gene) for 60 s at speed setting 6. All procedure was carried out using the Isolate II RNA Mini Kit (Bioline) following the manufacture’s protocol. Genomic DNA was removed from total RNAs by treating samples twice for 30 min with DNase I using the manufacturer’s protocol. Total RNA quality was checked by agarose gel electrophoresis and a Nanodrop ND-8000 (Thermo Scientific) was used to determine the concentration. 1 μg of total RNA was reverse-transcribed using an iScript™ cDNA Synthesis kit (Bio-Rad) according to the manufacturer's protocol. In the case of embryo and larvae incubated at different temperatures, total RNA (2 μg) from each sample was reverse-transcribed using the High Capacity cDNA Reverse Transcription Kit (Life Technologies) as previously described for openarray analysis [16, 26].

Real-time analysis (qPCR) was carried out on a CFX96™ Real-Time System (Bio-Rad). The qPCR assays were performed in duplicate in a 10 μL volume containing cDNA generated from 10 ng of the original RNA template, 300 nM each primer and 5μl of SsoAdvanced™ Universal SYBR Green Supermix (Bio-Rad). Primers to amplify choriolysins are depicted in Table 1. It should be note that set of primers SseHceaq and SseHcebq were designed on selected copy sequences of SseHCEa and SseHCEb tandem arrays, respectively and do not necessary amplify all the subunits in such tandem. In contrast, the SseHceglobal primers were located in conserved regions of SseHCEa and SseHCEb gene copies. The qPCR amplification protocol was as follows: 7 min for denaturation and enzyme activation at 95°C followed by 40 cycles of 30 s at 95°C and 1 min at 60°C. Data were normalized using the geometric mean of ubiquitin (*ubi*) and β-actin (*actb2*) [27] and the relative mRNA expression calculated using the comparative Ct method. The estimated amplification efficiencies for primer pairs were: 2.10 for SseHceaq, 1.86 for SseHcebq, 2.11 for SseHceboth, 2.16 for SseHEq, and 2.02 for SseLCEq.

### Collection and protein characterization of hatching liquid

To collect the batches of hatching liquid, fecundated eggs were incubated at an initial density of 7,000 embryos L^-1^ in 15-L cylinder-conical tanks with gentle aeration for 24h at 20–22°C until close to hatch. Then, eggs were filtered and concentrated at 50,000 eggs in 200 ml final volume with a gentle aeration. Larvae were monitored for one hour removing those hatched larvae by periodically filtering the sample throughout a 600-μm mesh. When most of the larvae were hatched, the hatching liquid was filtered by a 100μm mesh to remove any debris, aliquoted in 50 ml batches and kept frozen -20°C until use.

Protein concentrations in the hatching liquid were determined using Bradford method [28]. Samples were measured at 595 nm using a UV/Vis spectrophotometer (Perkin Elmer). The proteolytic activity was measured using the azocasein assay [29]. Briefly, the assay mixture (final volume 0.65 mL) contained: buffer (77 mM Tris–HCl, pH 8.5) and azocasein (7.7 mg/mL). The enzymatic assay was carried out at 37°C for 30 min, and the reaction was stopped by adding trichloroacetic acid (600 μL, 10% v/v), incubated on ice for 10 min and then centrifuged at 13,000 rpm for 10 min. A volume of 500 μl of supernatant was removed and added to an equal volume of 2M NaOH. The mixture was mixed and the absorbance was measured at 440 nm. One unit of enzyme activity was defined as the amount of the enzyme that resulted in an increase of absorbance of 0.01 (under the above assay conditions) at 440 nm.

Protein profile of the hatching liquid was characterized by SDS-polyacrylamide gel electrophoresis (SDS-PAGE) using a 12% gel in the presence of 2-mercaptoethanol according to the method of Laemmli [30]. Moreover, a zymography analysis was carried out to identify specific bands with proteolytic activity as previously described [31]. Briefly, samples were treated with Laemmli sampling buffer (without any reducing agent or boiling) and later loaded on polyacrylamide separating gel 8% (w/v) co-polymerized with 0.1% (w/v) casein and 4% (w/v) stacking gel. After electrophoresis, the gel was washed on an orbital shaker for 40 min at 37°C with 20 mM Tris buffer (pH = 8), containing 2% (v/v) Tween-20 and incubated overnight in 50 mM Tris buffer, 0.15 M NaCl, 10 mM CaCl_2_ and 7 mM NaN_3_ (pH = 8) at 37°C. The gel was stained using Coomasie Brilliant Blue R-250 and destained using an aqueous solution of 4% (v/v) methanol and 8% (v/v) acetic acid.

The effect of metal ions (Ca^2+^, Mg^2+^, Zn^2+^, and Cu^2+^) and protease inhibitors [phenylmethanesulfonyl fluoride and pepstatin A, ethylene diamine tetraacetic acid (EDTA)], on enzyme activity was assessed using the azocasein assay as described above. Concentrations tested are indicated in Table 2.

**Table 2:** Main features of choriolysin genes in Senegalese sole. The phylogenetic clade, presence of introns in gene structure, number of amino acids in the coding sequence (CS) and signal peptide (SP), molecular weight (kDa), unigene name (EST) in the SoleaDB, scaffold size (Scf Size) and chromosome position (Chr*) in *Cynoglossus semilaevis*.

	Clade	introns	CS	SP	kDa	EST	Scf Size	Chr*
SseHCEa	I	No	259	18	29.7	unigene11597	581.5 kb/	5
SseHCEb	I	No	259	18	29.8	unigene546404		
SseHE	IIa	7	267	20	30.3	unigene331028	67.5 kb	14
SseLCE	IIb	7	276	23	31.7	unigene228567, unigene53188	285 kb	7

## Human skin cell culture and cytoxicity tests

Primary Normal Human Dermal Fibroblasts (NHDF) isolated from normal human adult skin were purchased from Lonza Clonetics™ (Lonza Walkersville, USA) and cultured as previously described [32, 33]. The cell proliferation test was assessed by using an MTT kit (Vybrant MTT cell proliferation assay kit, Thermo Fisher Scientific) according to the manufacture's protocol. NHDF were incubated for 24h with serial dilutions (from 1 to 10^−5^) of a dialyzed hatching liquid batch containing an activity of 9.2 U mL^-1^ and optical density measured at 570 nm.

### Statistical analysis

For qPCR analyses during embryo development, one-way ANOVA was carried out followed by a Tukey post-hoc test (*P < 0.05). To test the differences associated with incubation temperatures, a Kruskal-Wallis ANOVA and post hoc Dunn's test was performed. Data are presented as mean ± standard error of the mean (SEM). Statistical analyses were carried out using SPSS v21 software (IBM).

## Results

### Choriolysin identification and genomic structure

Four putative expressed sequence tags (ESTs) encoding for choriolysins were identified after searching at the SoleaDB [22] named as SseHCEa, SseHCEb, SseLCE and SseHE. The open reading frames ranged between 259 and 276 codons (Table 2). Amino acid identity between the paralogs ranged between 52.9 and 54.8% except between SseHCEa and SseHCEb copies that was higher than 96.5%. Protein sequence analysis identified a putative signal peptide in the four genes ranging from 18 to 23 residues in length (Table 2 and S1 Fig) followed by a putative propeptide of 49 amino acids (predicted as a 3vtg domain) just after the C-terminal of the signal peptide. All the four predicted matured enzymes had the consensus active site sequence of the astacin family protease, HExxHxxGFxHExxRxDR, the conserved sequence SXMHY and six conserved cysteine residues specific for fish hatching enzymes (S1 Fig). The amino acid sequences of SseHCEa, SseLCE and SseHE were submitted to SWISS-MODEL and the three-dimensional structure of protein was constructed (S1 Fig). The three proteins had a highly conserved tertiatry structure was well as the binding site to Zn^2+^. SseHCEa showed the highest identity (65.8%) with the crystal structure (3vtg.1) of the high choriolytic enzyme 1 (HCE-1) from medaka (*Oryzias latipes*). The SseLCE and SseHE showed the highest identity (57.6 and 57.3%, respectively) with the high choriolytic enzyme 1 (3lqb.1) from zebrafish (*Danio rerio*).

**Fig 1:**
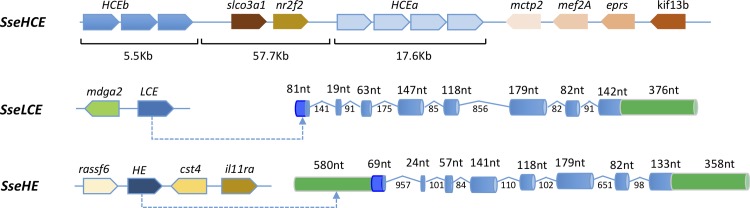
Synteny and gene structure of SseHCE, SseLCE and SseHE choriolysins in Senegalese sole. Scaffolds containing the chorylysins were blasted onto NCBI to identify genes and later validated with genomicus database. Each gene is represented by an arrow in differnt colors within each cluster. The orientation indicate the transcription direction. SseHCE paralogs (a and b) clustered in the same scaffold and the size of genomic regions in kilobases are shown. In SseLCE and SseHE, the gene structure and including length of introns and exons is shown. The 5' and 3'-UTR are indicated in green and the signalIP in dark blue. Gene names: *cst4*, cystatin-S-like; *eprs*, glutamyl-Prolyl-TRNA Synthetase; *il11ra*, interleukin-11 receptor subunit alpha; *kif13b*, kinesin family member 13B; *mctp2*, multiple C2 and transmembrane domain containing 2; *mef2A*, myocyte enhancer factor 2A; *nr2f2*, COUP transcription factor 2; *slco3a1*, solute carrier organic anion transporter family member 3A1; *rassf6*, association domain-containing protein 6.

The genomic structure of these four enzymes was investigated by blasting the sequences onto an assembled genome draft of S. *senegalensis*. SseHCEa and SseHCEb were located within the same scaffold that mapped onto the chromosome 5 of *Cynoglossus semilaevis* (Fig 1). These genes were tandemly arrayed into two clusters 47.5 kb apart and separated by the solute carrier organic anion transporter family member 3A1 (*slco3a1*) and COUP transcription factor 2 (*nr2f2*). Gene copies of both SseHCE arrays had no introns and main polymorphisms were located in the 3'-UTR. A set of four genes (*mctp2 mef2A eprs* and *kif13b*) upstream SseHCEa was highly conserved in other teleosts (Fig 1). The scaffold containing the SseLCE blasted onto the chromosome 7 of *C*. *semilaevis* and the gene was structured in 8 exons and 7 introns contiguous to the MAM domain containing glycosylphosphatidylinositol anchor 2 (*mdga2*) gene. The SseHE also contained 8 exons and 7 introns and was flanked by ras association domain-containing protein 6 (*rassf6)* and the cystatin-S-like (*cst4*) and interleukin-11 receptor subunit alpha (*il11ra*) genes in a scaffold that blasted onto the chromosome 14 of *C*. *semilaevis* (Fig 1).

### Phylogeny

**Fig 2:**
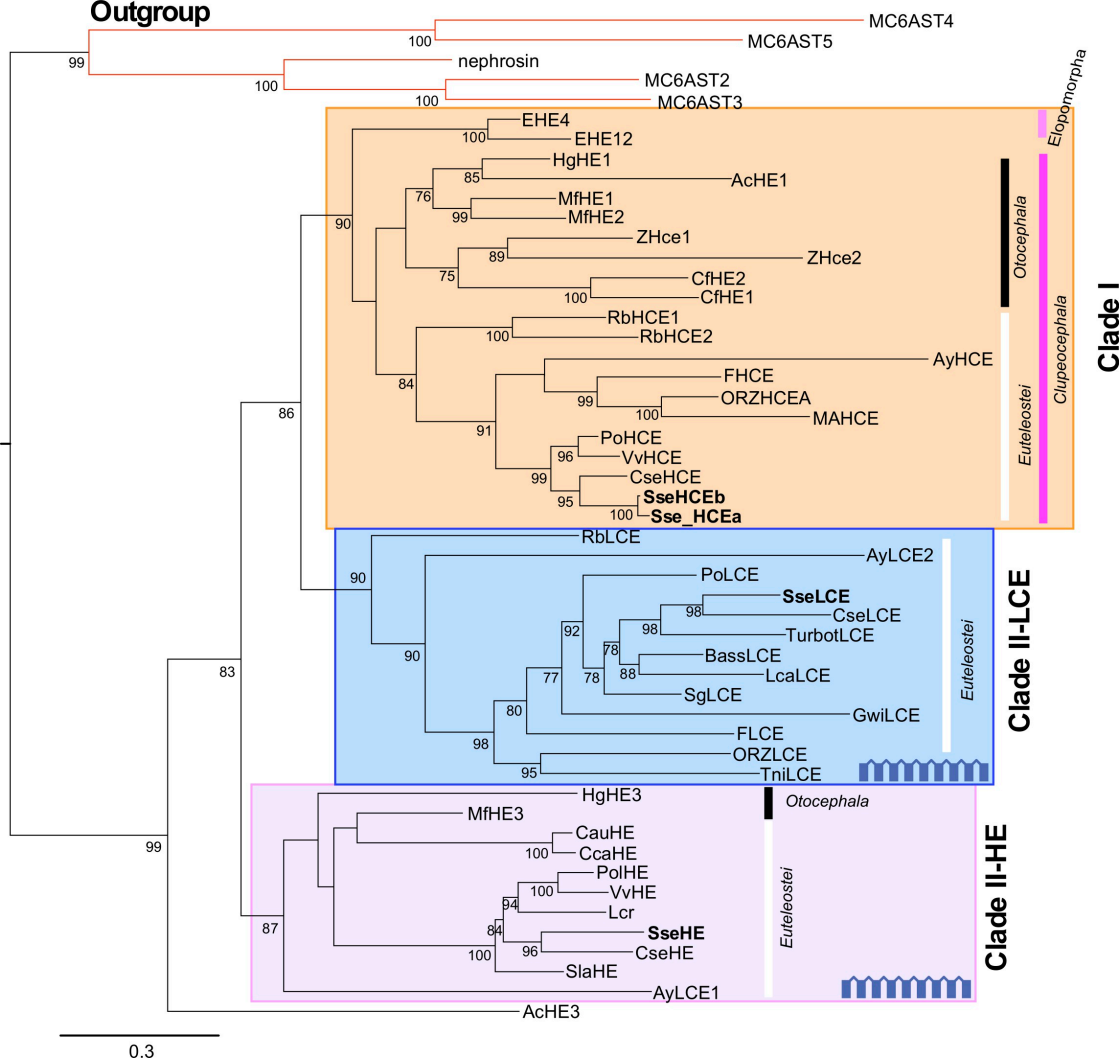
Phylogenetic relationships among the predicted sequences of Senegalese sole choriolysisn and the corresponding deduced amino acid sequences from other fish using the Maximum Likelihood method. The clade-I and the subclades HE and LCE in clade II are indicated. Astacin family metallo-proteases from *Oryzias latipes* were used as outgroup to root tree. Only bootstrap values higher than 70% are indicated on each branch. The scale for branch length (0.3 substitutions/site) is shown below the tree. *Solea senegalensis* sequencies are in bold. Species abbreviations are indicated in S1 Table.

Phylogenetic analysis indicated that choriolysins could be divided into two main clades named as I and II that grouped the HCE- and LCE-like enzymes, respectively (Fig 2). The clade I contained the SseHCEa and SseHCEb paralogs as well as the high choriolytic enzymes of a diverse range of Elopomorpha and Clupeocephala species. The clade II was structured into two well-supported subclades named as LCE and HE according to the nomenclature previously established [8]. The subclade LCE grouped the low choriolytic enzymes in Otocephala and Euteleostei. The subclade HE contained only hatching enzymes from Euteleostei. These two subclades were highly conserved in flatfish (Fig 2).

### Expression profiles in embryos and larvae

**Fig 3:**
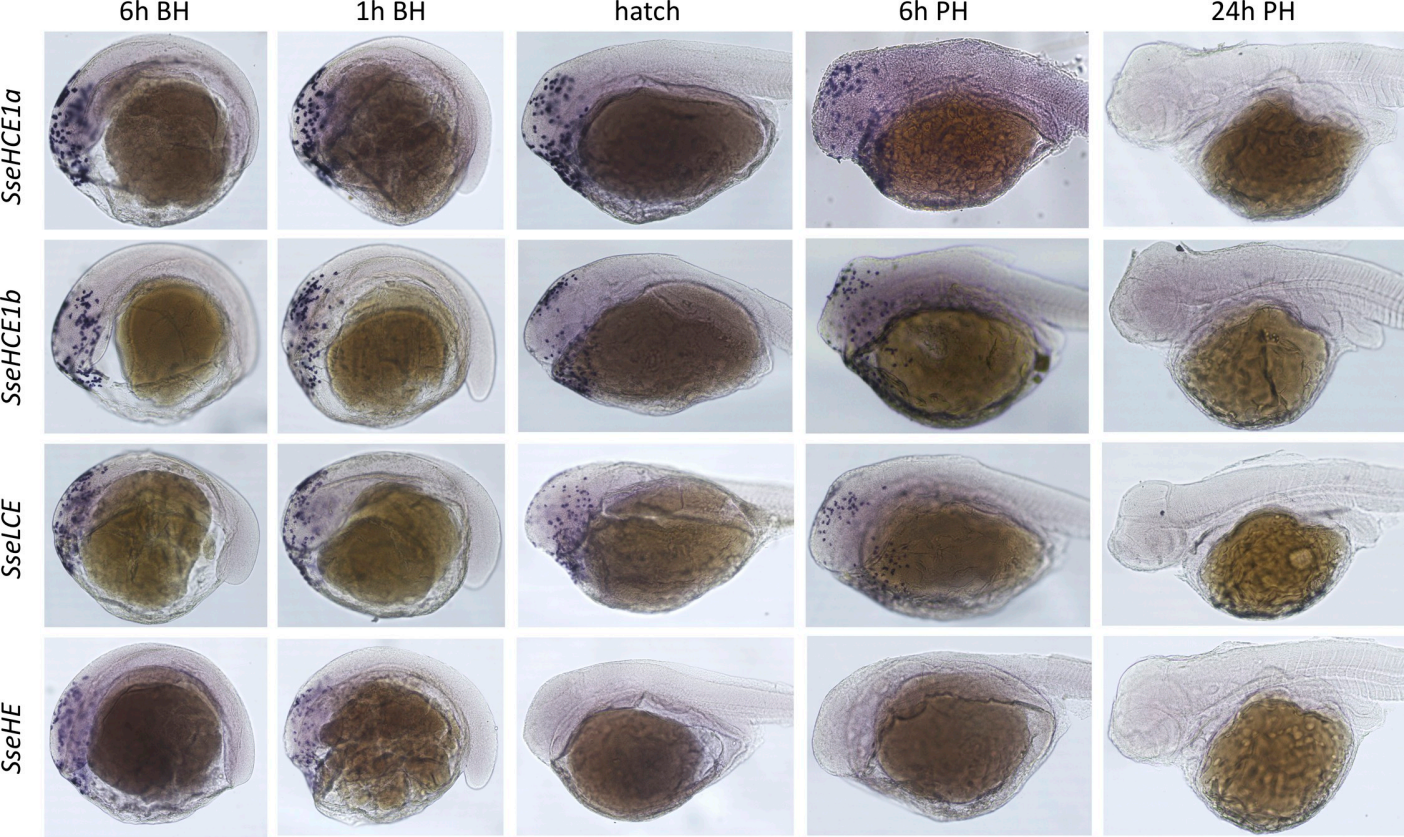
WISH of chorilysins in sole at 6 and 1h before hatching, at hatch and 6h after hatching. Lateral views are for the four paralogs are presented.

To establish the expression profiles of the four putative choriolysin genes in embryos around the hatch, a set of samples from 6 h before hatching until 6 h post-hatch were selected for *in situ* whole-mount hybridization (WISH) (Fig 3). Hybridization signals for the four genes were mainly located around the forebrain and optic vesicles with a bilateral pattern mostly evident in SseHCEb at 6 h before hatching (Figs 3 and S2). At 1h before hatching and at hatch, the signals spread toward the anterior surface of the yolk sac with SseHCEa, SseHCEb and SseLCE positive signals still observable in the head. No signal was detected for SseHE from hatching. SseHCEa, SseHCEb and SseLCE showed weak signals at 6h post-hatching. No expression signal was evident at 24h post-hatch (S2 Fig).

**Fig 4:**
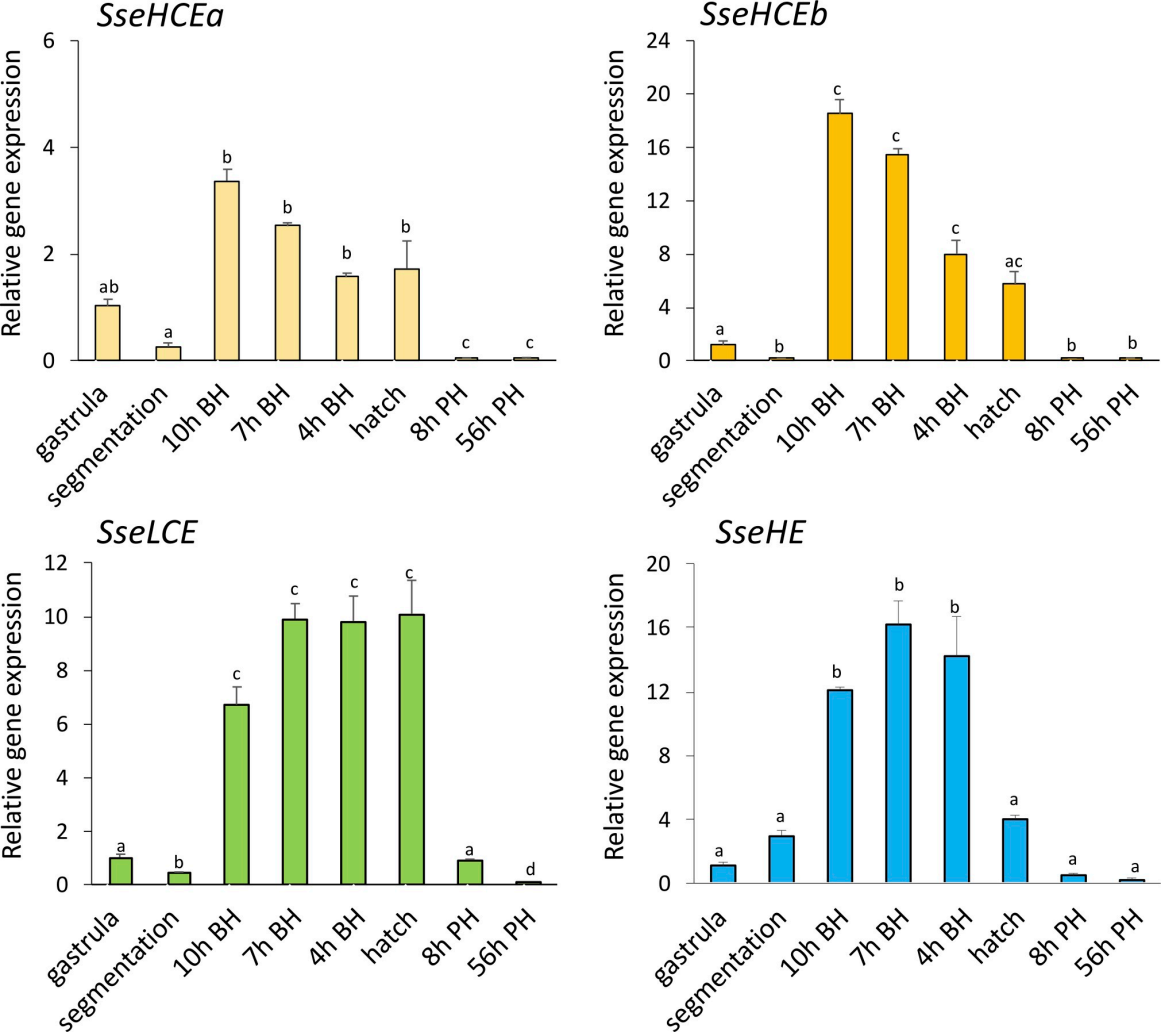
Relative expression levels of the SseHCEa, SseHCEb, SseLCE and SseHE paralogs in gastrula, segmentation, at 10, 7 and 4h before hatching (BH), at hatch and 8 and 56h post-hatch (PH). Data were expressed as the mean fold change (mean + SEM, n = 3) from the calibrator group (gastrula). Different letters denote significant differences among samplings (*P*< 0.05).

In addition to WISH, the mRNA levels of the four choriolysins from gastrula to 56 h post-hatch were quantified by qPCR. All genes had detectable mRNA levels from gastrula stage but the transcript amounts highly increased at 8h before hatching and maintained elevated until larval hatch (Fig 4). Thereafter, the expression levels dropped to recover the steady-state levels.

**Fig 5:**
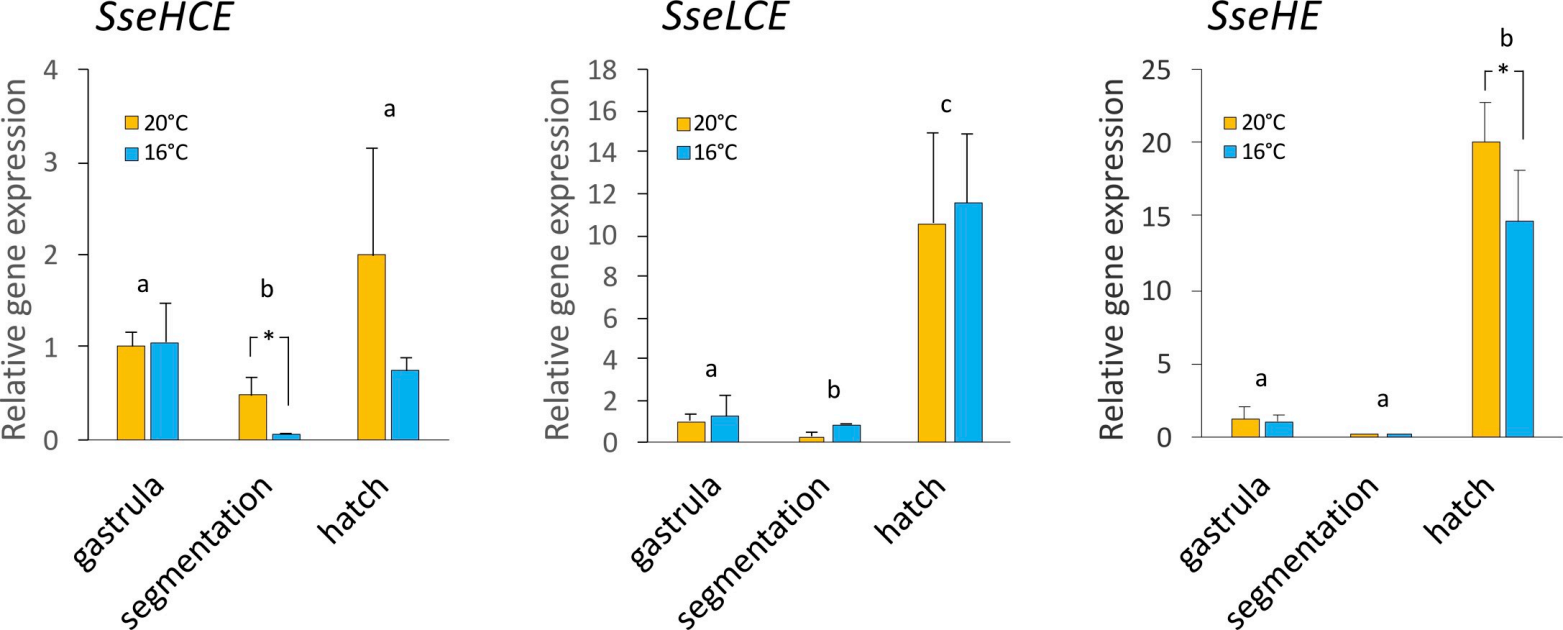
Relative expression levels of the SseHCE, SseLCE and SseHE genes in gastrula, segmentation and at hatch in embryos. Data were expressed as the mean fold change (mean + SEM, n = 3) from the calibrator group (gastrula). Different letters denote significant differences among stages (*P*<0.05). The asterisks indicate differences between thermal treatment in a specific developmental stage.

To evaluate if expression patterns in clade I and clade II choriolysins were modulated by temperature, embryo samples cultivated at 16 and 20°C were analyzed from gastrula stage until after hatching. The three genes activated the expression just at hatch. The SseLCE was not significantly affected by temperature (Fig 5). However, the SseHCE paralogs and the SseHE showed statistically significant lower mRNA levels at 16°C than 20°C in the segmentation stage and at hatch, respectively. Comparison of mRNA levels between choriolysins indicated that SseHCE transcripts were more abundant than of LCE and HE at hatch (11.3- and 6.9-fold-higher, respectively assuming no differences in amplification efficiency and relative sensitivity factor K_RS_ [34]).

### Hatching liquid characterization

**Fig 6:**
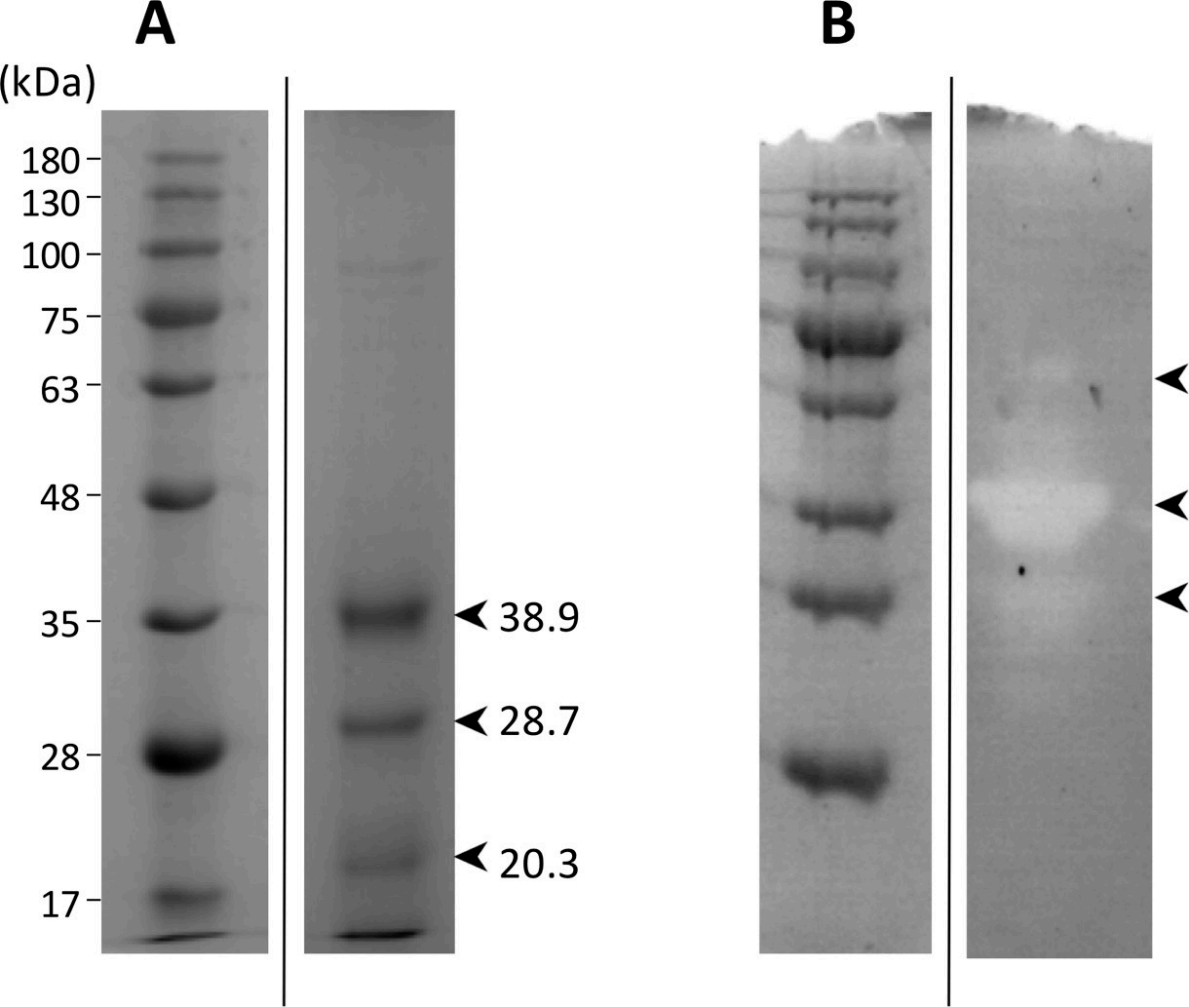
A) SDS-polyacrylamide gel electrophoresis (PAGE) of the hatching liquid. The figures on the left refer to the molecular weights (kDa) of the markers. The bands identified and the expected molecular size as determined by Fiji software are indicated. B) Casein Zymography. Bands with activity are indicated (arrow heads). All hatching liquid samples shown were previosly dialyzed. The ladder and the samples in SDS-PAGE and zymography were run in the same gel with other additional samples but they were omitted to show only the selected samples (see S1 raw images). This fact is indicated by a vertical black line between the ladder and the sample.

SDS-PAGE analysis of hatching liquid identified three major bands of 20.3, 28.7 and 38.9 kDa (Fig 6A). Although some variations in the band patterns were observed between some hatching liquid batches, these major bands were constantly observed (S3 Fig). The casein zymography clearly identified one strong and two secondary weak proteolytic zones. The use of a buffer containing Ca^+2^, resulted in the enhancement of the proteolytic activity (Fig 6B).

**Fig 7:**
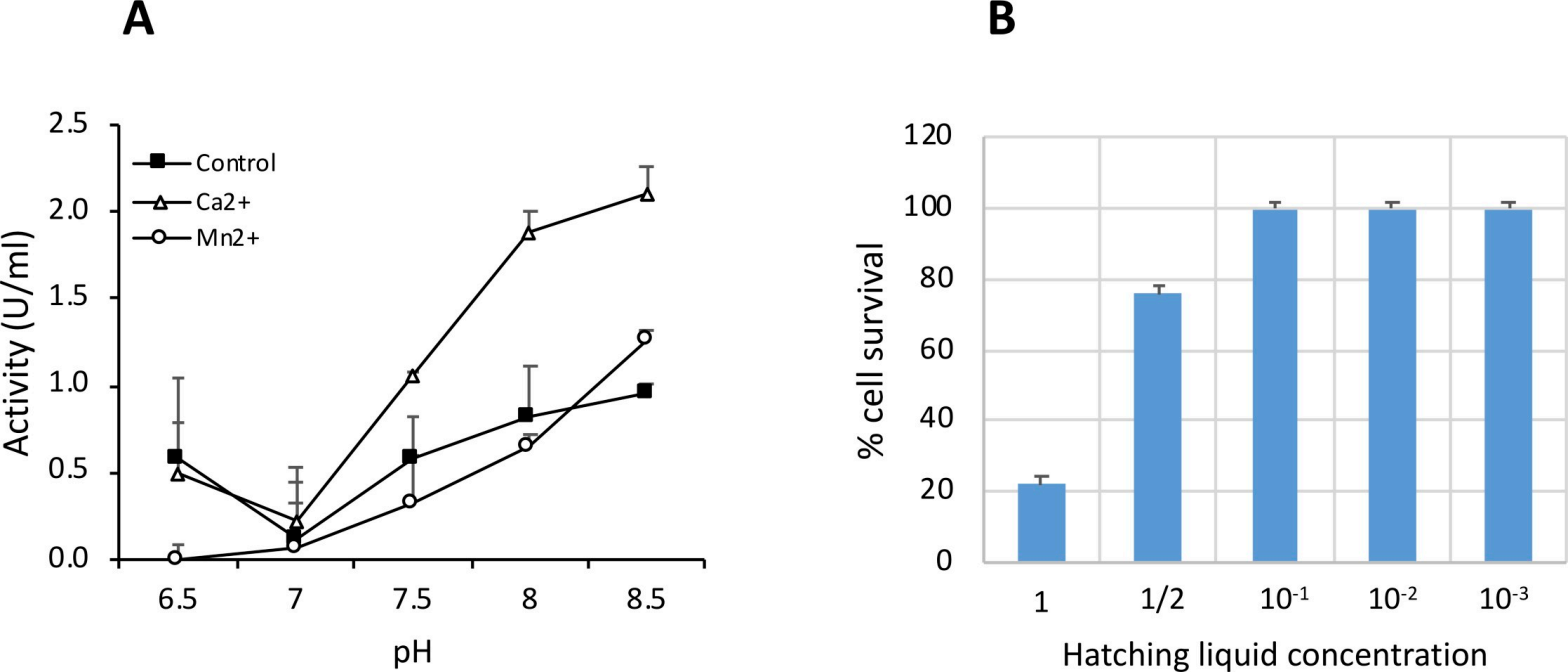
A) Proteolytic activity of hatching liquid as function of pH and metals (Ca^2+^ and Mn^2+^). The caseinolytic activity was calculated with azocasein as a substrate. B) Toxicity of hatching liquid in human fibroblast cells as determined by MTT. Dilutions of a dialyzed batch are indicated.

The effects of some metal ions and protease inhibitors are depicted in Table 3. Proteolytic activity was strongly inhibited by EDTA, Zn^2+^, Cu^2+^ and Co^2+^. In contrast, activity was enhanced by the addition of Ca^2+^ and Mg^2+^. No effect of PMSF and pepstatin A on proteolytic activity was observed. The recovery of enzyme activity in samples previously inhibited by the chelator EDTA indicated that the Ca^2+^ was the most effective metal. The optimal pH for protease activity in the hatching liquid was 8.5. The addition of Ca^2+^ but not Mn^2+^ clearly enhanced the activity (Fig 7A).

**Table 3:** Proteolytic activity of hatching liquid. Upper part, inhibition/enhancement assays with different metals, the ion chelator EDTA and the serine peptidase inhibitors PMSF and pepstatin A. Lower part, recovery trial in EDTA-inhibited assays added metals. Data are shown as % with respect to the crude hatching liquid. Concentrations used for each compound in the assays are indicated.

		Concentration	Proteolytic activity (%)
Inhibition-Enhancement assays	Ca^2+^	1mM	187±53
	Mg^2+^	1mM	184±89
	Mn^2+^	1mM	112±29
	Fe^2+^	1mM	74±14
	Co	1mM	42±12
	Cu^2+^	1mM	46±12
	Zn^2+^	1mM	32±14
	EDTA	20mM	23±23
	PMSF	2mM	86±9
	Pepstatin A	0.5mM	108±24
Recovery assays	HL		100
	HL+EDTA	20mM EDTA	19±4
	HL+EDTA+Ca^2+^	20mM EDTA+ 10mM Metal	168±8
	HL+EDTA+Mg^2+^		59±6
	HL+EDTA+Cu^2+^		35±17
	HL+EDTA+Zn^2+^		24±6

To test the effect of hatching liquid on fibroblasts, the cytotoxic of a batch with 9.2 U/mL enzyme activity was tested (using serial dilution from pure to 10^−4^) in the primary human dermal cell cultures NHDF using the MTT assay (Fig 7B). Pure (low viability 20%) and 1/2 dilution (76%) impaired cell viability but dilutions containing less than 0.9 U/ml resulted in 100% cell viability.

## Discussion

Choriolysins are essential proteases in fish life cycle controlling the transition from embryo to larval stage. The tight control of these developmental stages for larval survival exerts a high evolutionary pressure on their functional domains resulting in a high conservation in teleost lineage [1, 3]. In spite of these functional evolutionary restrictions, choriolysin-encoding genes irradiated in the fish genomes as an adaptive mechanism to different environments that can be present in a variable number of copies in some species as the result of two major forces: the genome duplication that occurred in teleost lineage and the retrocopy and translocation events that modified the intron-exon structure and genome organization [5, 7, 35, 36]. In the flatfish *S*. *senegalensis*, four genes located in three different genomic scaffolds were identified. These four genes had highly conserved the key domains of astacin metalloproteases responsible for enzymatic activity and structure such as the HExxHxxGFxHExxRxDR involved in the zinc-binding ligands, the methionine turn SxMHY required for the structural integrity [1, 3, 10] and the six conserved cysteines present in hatching enzymes responsible for the stabilization of tertiary structure [36]. Moreover, predicted three-dimensional structures were highly conserved supporting that they were functional metalloproteases.

Choriolysin-encoding genes are routinely classified into two main clades, referred to as I and II that act cooperatively for the stepwise cleavage of the egg envelope proteins. While enzymes in clade I swells the envelope, the clade II choriolysins fully solubilize the chorion and release the larvae [5, 7]. In this study, two type I HCE genes tandemly arrayed in separate multicopy clusters and two type-II enzymes, named HE and LCE, were identified. Lack of introns and tandemly arrangement in the genome is a common feature for HCE genes in euteleostei. These characteristics are mainly explained by a retrocopy origin in which the new genes maintained the upstream promoters and rearranged through the genome in functional multicopy arrays [7, 35]. Although the HCE clusters moved at least twice in fish genomes and synteny is conserved in euteleostei [7], a further reorganization seemed to occur in the flatfish lineage to create two separate gene clusters (referred to as HCEa and HCEb). Although HCEa and HCEb open reading frames were highly similar, some divergence was observed in the non-coding 3'-UTR that indicating that these gene rearrangements occured recently although currently they could be not evolving concertedly.

In addition to the particular organization of HCE clusters in the genome in *S*. *senegalensis*, this study also demonstrated that clade II contained two well-supported HE and LCE subclades as previously identified in other flatfish species [8]. While the SseLCE is widely detected in Euteleostei, the SseHE was detected in Otocephala and a reduced number of euteleosteans. Moreover, these two genes were structured in 8 exons and 7 introns in different scaffolds indicating that they were derived from the ancestral copies in basal actinopterygians [37]. Previous studies that evaluated functional characteristics of enzymes belonging to these two subclades indicated that they could have followed a neofunctionalization acquiring different cleavage activities [6]. The conservation of SseHE and SseLCE in *S*. *senegalensis* could represent a protease activity specialization as an evolutionary co-adaptation to lead to complete solubilization of envelopes and achieve optimal hatching rates.

Expression patterns of the four genes identified in *S*. *senegalensis* indicated that a high expression in the hours previous to the larval hatch and they appeared located in the hatching gland cells spread through the yolk sac although with in intensity and temporal differences. The highest expression of HCE genes seems to be a conserved feature in euteleostei [5, 8] due to the lack of introns and the multicopy gene structure that are two adaptive mechanism able to enhance the clade-I enzyme abundance and envelope swelling [5]. It is interesting that SseHE were hardly detected at hatch by WISH while SseHCE paralogs and SseLCE showed longer expression patterns suggesting that these latter two genes could play a major role in sole hatching. The earlier decline in SseHE transcript were also observed in the closely-related flatfish *Solea solea* with high expression of HCE at 1 dph that decline at 4 dph but without changes in the HE steady-state levels [38]. It should be noted that some variation in the SseHE mRNA levels in larvae at hatch between experiments was observed that could represent temporal differences in the framework of larval sampling times after hatching. Also, the distribution pattern of hatching gland cells in the yolk sac fits better to the hole- than rim-hatching model as previously reported in flatfish with small-sized eggs as it is the case of sole [8, 12]. Although the incubation time was hypothesized as a factor influencing the levels of hatching enzyme as they accumulate in longer incubations periods [8], our data indicate that a delay in development as occurs at low incubation temperatures slightly modifies expression patterns with a decrease in expression levels and identifies the envelope characteristics and egg structure as the main factor shaping evolution and expression profiles.

Analysis of hatching liquids confirmed a major band with proteolytic activity that matched with expected sizes. Moreover, the maximal proteolytic activity was in the pH range 8–8.5 as previously reported for purified choriolysins in fish [39–41]. Moreover, the strong inhibition of the metal chelator EDTA and the slight effect of serine protease inhibitors (PMSF and pepstatin A) on the proteolytic activity, indicated that the proteases in the liquid hatching belong to the metalloprotease family. Analysis of the effect of metal ions on enzyme activity suggested that the Zn^2+^, Cu^2+^, and Co^2+^ act as inhibitors and Cu^2+^ and Mg^2+^ as enhancers of the proteolytic activity. Moreover, Ca^2+^ was the most effective metal ion that rescued the enzyme activity, previously inhibited by EDTA. The calcium ion has been related to enzyme stabilization in the astacin family [3]. Previous studies in medaka using purified HCE and LCE enzymes indicated that these choriolysins are strongly dependent on Ca^2+^ followed by Mg^2+^ and Zn^2+^ [40, 41]. The addition of Ca^2+^ and Mg^2+^, but not Zn^2+^ or Cu^2+^, enhanced the proteolytic activity of purified choriolytic enzymes in the flatfish Japanese flounder [42]. However, the Zn^2+^ was the most effective metal ion towards the EDTA-inhibited enzymes in medaka [40, 41]. The lack of response of Zn^2+^ in our samples to overpass the inhibitory effect of EDTA could be due to a dose effect as also reported in other members of astacin family [43] or the use of a complex hatching liquid instead of purified enzymes that could modify Zn^2+^ availability. Both of these hypotheses require further validation. Moreover, the effect of hatching liquid on fibroblast cells pinpoints a potential use of hatching liquid batches for cosmetic applications particularly for moisturizing and skin exfoliation.

In conclusion, four choriolysin genes were identified in *S*. *senegalensis*. The genomic analysis indicated that the HCE paralogs might be divergently evolving in flatfish genomes due to rearrangement of multicopy clusters and synteny that could interfere in concerted evolution mechanisms. Expression analysis indicated that most of the enzymes are highly expressed before hatching distributed in the yolk sac indicating a hole-induced hatching model. In spite of the synchronized induction at hatch, choriolysins displayed differences in transcript abundance and temporal expression profiles indicating specific actions to dissolve the egg envelope. The analysis of hatching liquid confirmed the size of proteases and that they were metalloproteases whose activity could be enhanced mainly by adding Ca^2+^. The results are relevant to sole aquaculture but also open a new field for circular economy by promoting the use of hatching liquid in hatchery as a sustainable source of proteases in non-aquaculture fields.

## Supporting information

S1 Table Sequences used in phylogenetic analysis. The taxonomic order, species name, common name, gene name and Accession numbers are indicated.
(DOC)

S1 File Choriolysin sequences of clones used as probes in WISH analysis. (DOCX)

S1 Fig A) Multiple alignment of amino acid sequences of EHE12 (*Anguilla japonica*) and SseHCEa, SseHCEb, SseLCE and SseHE choriolysins. The signal peptide and propeptide sequences are boxed in light and dark grey, respectively. Dashes and dots represent gaps and identity with respect to EHE12, respectively. Conserved cysteines are indicated in green and the zinc binding motif in light orange. The Met-turn sequence is underlined. B) Three-dimensional structure of SseHCEa, SseLCE and SseHE proteins.
(PDF)

S2 Fig WISH analyses for SseHCEa, SseHCEb, SseLCE and SseHE choriolysins using the sense and antisense probes. The lateral, ventral and dorsal views are shown for antisense probes.
(PDF)

S3 Fig SDS-Polyacrylamide gel electrophoresis (PAGE) of a hatching liquid batch showing a complex band pattern. The figures on the left refer to the molecular weights (kDa) of the markers.
(PDF)

S1 raw images Original blots. Loading order, sample identifiers, method used to capture the image, and the the selected lanes from that original image are indicated.
(PDF)
