# Supplementary material for: Genomic and phylogenetic analysis of choriolysins, and biological activity of hatching liquid in the flatfish Senegalese sole
Source: PLoS One. 2019 Dec 5;14(12):e0225666. doi: 10.1371/journal.pone.0225666 (PMC6894847; doi:10.1371/journal.pone.0225666)
Supplement: S1 Table — The taxonomic order, species name, common name, gene name and Accession numbers are indicated. (DOC) [file pone.0225666.s001.doc]

| Classification | Species | Name in this study | Gene name | Accession No. |
| --- | --- | --- | --- | --- |
| Elopomorpha |  |  |  |  |
| Anguilliformes | *Anguilla japonica* | Japanese eel | EHE4 | AB071423 |
|  |  |  | EHE12 | AB071427 |
| Otocephala |  |  |  |  |
| Clupeiformes | *Clupea pallasii* | Pacific herring | HgHE1 | AB433584 |
|  |  |  | HgHE3 | AB433586 |
|  | *Engraulis japonicus* | Japanese anchovy | AcHE1 | AB433587 |
|  |  |  | AcHE3 | AB433589 |
| Gonorynchiformes | *Chanos chanos* | Milkfish | MfHE1 | AB480009 |
|  |  |  | MfHE2 | AB480010 |
|  |  |  | MfHE3 | AB480011 |
| Cypriniformes | *Danio rerio* | Zebrafish | ZHce1 | AB175621 |
|  |  |  | ZHce2 | AB175620 |
|  | *Carassius auratus* | Goldfish | CauHE | XP_026051816 |
|  | *Cyprinus carpio* | Common carp | CcaHE | XP_018919344 |
| Siluriformes | *Silurus asotus* | Catfish | CfHE1 | AB480016 |
|  |  |  | CfHE2 | AB480017 |
| Euteleostei |  |  |  |  |
| Salmoniformes | *Oncorhynchus mykiss* | Rainbow trout | RbHCE1 | AB480022 |
|  |  |  | RbHCE2 | AB480023 |
|  |  |  | RbLCE | AB480024 |
| Osmeriformes | *Plecoglossus altivelis* | Ayu | AyHCE | AB256940 |
|  |  |  | AyLCE1 | AB256941 |
|  |  |  | AyLCE2 | AB256942 |
| Perciformes | *Dicentrarchus labrax* | European seabass | BassLCE | LC337380 |
|  | *Lates calcarifes* | Asian seabass | LcaLCE | XM_018696137 |
|  | *Larimichthys crocea* | Yellow croaker | LcrHE | XP_027134478 |
|  | *Seriola lalandi dorsalis* | Yellowtail amberjack | SlaHE | XP_023263038 |
| Scorpaeniformes | *Setarches guentheri* | Channeled rockfish | SgLCE | AB353107 |
| Blenniiformes | *Gouania willdenowi* | Blunt-snouted clingfish | GwiLCE | XM_028439401 |
| Tetraodontiformes | *Tetraodon nigroviridis* | *Tetraodon* | TniLCE | ENSTNIT00000014103 |
| Pleuronectiformes | *Paralichthys olivaceus* | Japanese flounder | PoHCE | AB480031 |
|  |  |  | PoLCE | AB480032 |
|  |  |  | PolHE | AB898066 |
|  | *Verasper variegatus* | Spotted halibut | VvHCE | AB898060 |
|  |  |  | VvHE | AB898062 |
|  | *Scophthalmus maximus* | Turbot | turbotHCE | ENSSMAG00000006890f |
|  | *Cynoglossus semilaevis* | Tongue sole | CseHCE | XM_008337088 |
|  |  |  | CseLCE | ENSCSET00000002810 |
|  |  |  | CseHE | XM_017038974 |
|  | *Solea senegalensis* | Senegalese sole | SseHCEa | MK789472, MK789473 |
|  |  |  | SseHCEb | MK789469-MK789471 |
|  |  |  | SseLCE | MK789467 |
|  |  |  | SseHE | MK789468 |
| Cyprinodontiformes | *Fundulus heteroclitus* | Killifish | FHCE | AB210813 |
|  |  |  | FLCE | AB210814 |
| Beloniformes | *Oryzias latipes* | Medaka | ORZHCEA | M96170 |
|  |  |  | MAHCE | AB256944 |
|  |  |  | ORZLCE | M96169 |
|  |  |  | MC6AST2 | AB256946 |
|  |  |  | MC6AST3 | AB256947 |
|  |  |  | MC6AST4 | AB256948 |
|  |  |  | MC6AST5 | AB256949 |
|  |  |  | nephrosin | AB256945 |
