# Supplementary material for: Genomic and phylogenetic analysis of choriolysins, and biological activity of hatching liquid in the flatfish Senegalese sole
Source: PLoS One. 2019 Dec 5;14(12):e0225666. doi: 10.1371/journal.pone.0225666 (PMC6894847; doi:10.1371/journal.pone.0225666)
Supplement: S1 File — (DOCX) [file pone.0225666.s002.docx]

>LCEprobe6.3

ACGGATGCTCCTCTTTGCTGGGTTACACCGGAGACAAGCAGGTGCTGTCACTGCAGAGATACGGCTGCATAAACAACGGCATCATCCAGCACGAGATGCTGCACGCGCTGGGCTTCTACCACGAACACACCCGCAGTGACCGCGACCAGTACATCAGGATCAACTGGGAAAACATAAATAAATATTTTGTCTACAACTTCCGTAAAATGGACACGGATAATCTCAACACTCCGTATGACTATTCCTCTGTGATGCACTACGGAAGAACTGCCTTTGGGACGCAGCGCCAAGAAACTATAACTCCAACCTACGACCCGTCTGCTACCATCGGGCAGAGGGAGGGTTTGTCCAACATCGACATTTTCAGGATCAACAAGCTCTACAGGTGCTGGGGCTTCAGT

>SseHEprobe2.2

CTCCTCTCTGCTGTCTTCACCGTCCTGCTGGGGTTTTCAGCACAAAGCCACTCACTGGTGCGATCTCATGATGAAGGTGTAGATCCCGATCCATCTCAACGTTTGGACATAACTTCAAGAATCCTGGGAGCAAATAGACGTGTGAGTCAAATTCTGGTGGAAGGCGATGTCACAATTTCTAAGACAAGGAATGCCATGAAATGCTGGGGCGGCTACTGCACGTGGAAGAAATCCTACAACGGACTTGTTGAAGTGCCATATACCATCAGTGATTATTACTACGACAGCGAAAAAGCCTCAATTCTGAAAGCCATGGAAACTCTCCACCAGAAGACCTGTGTTCGCTTTGTTCCCCATCGAGGCCAGACTGACTACCTGAGCATCGAGAGTGAACTCGGGTGCTGGTCCTCTGTTGGCAGAGACGGGGGACATCAGGTGGTGTCTCTGTCCGTTTACGGTTGTCTCGAACACGGGACCATTCAGCACGAGCTCCTCCACGCGCTGGGCTTCCACCACGAGCACACGAGGAGCGACAGGGAC

>HCEa4.2

ACAACTTCTACAAGCAGAACACCAACAACCTGAACACTCCCTACGACTACACCTCCGTCATGCACTATGGAAGAACAGCCTTCACTGTCCAGTACGGAAGGGATACAATCACTCCCATCCCCAACCCCAATGTCCAGATCGGCCAGAGGCGGGGCATGTCCCGCTGGGACATCATCAGGATCAACGCCCTCTATGGCTGCTAATAATGCTAATGTTAAATGTCCTGTTTGTGTTCCTTTTTTTCTGTCATTAATAAAAAGCAACTATATTCTATGATCCAAATGTTTAATTTAACTACATTCTTTTTGAATTCTGATAACTCAATATATGACCACAACATGCCTCCAATGTCCAAAATAATGGTAAATCATCACGACTGTTGTTGTATCAGTTTTATCTATTACAAAAATCTTCCACATGAAGTCATTTACACAAACACACAGTCACAGAGGCAAATAAAGGATTGCTAAAGCTGCATGAATGTCTGTTG

>HCEb8.3

ACAACTTCTACAAGCAGAACACCAACAACCTGAACACTCCCTACGACTACTCCTCCGTCATGCACTATGGAAGAACAGCCTTCACTGTCCAGTACGGGAGAGATACAATCACTCCCATCCCCAACCCCAATGTCCAGATCGGCCAGAGGCGGGGCATGTCCCGCTGGGACATCATCAGGATCAACGCCCTCTATGGCTGCTAATAATGCTAAAGTTAAATGTCCTGTTTGTGTTCTTTTTATCCTGTCATTAATAAAAGCAACTATAATCTATGATAAACAATGGTCAAGTTTACTACAGAATATATGACCCCAACAAATGTCAAAAATAAAGGTAAAACAACATGACTGCTATTGTCATTTTTAGGTGACTGTGTTTGAGATTACAAAATACTTTCATATATACATGAAGTCATCTTCACCTAAATACAATCACTGAGCAAAGGAAGAATGGCC
