# Supplementary material for: Genomic and phylogenetic analysis of choriolysins, and biological activity of hatching liquid in the flatfish Senegalese sole
Source: PLoS One. 2019 Dec 5;14(12):e0225666. doi: 10.1371/journal.pone.0225666 (PMC6894847; doi:10.1371/journal.pone.0225666)

A)

```
                 10        20        30        40        50        60        70        80        90       100
EHE12    MD----QRLISTILALLLSLSLAHHVEDLESEDANEIEDPDDLDITTRIMQSNNGSIEMLMEGDLLVSNTRNAMKCRNN--RCLWKKSSNGLVEVPYTVS
SseHCEa  .T----PT-V.LL.L...G..R..PLH.------EGN..E..V......LT...ATD.I.L.....APK.......WYQ--S.Q...GY....TI.F...
SseHCEb  .T----PT-V.LL.L...G..R..PLH.------E.N..E..V......LT...ATD.I.L.....APK.......WYQ--S.Q...GN....TI.F...
SseLCE   ..FNTSTSVPLLL.L...GF.K..DDN.QGAVHDLSMDQSAKE.F.AT.LRM....SDF.L...VMIP.......I.EPYS..P..A..Y..I.FIL.
SseHE    ..----LT.L.AVFTV..GF.AQS.SLVRSHDEGVDPDPSQR....S..LGA.RRVSQI.V...VAI.K.......WGG--Y.T....Y.........I.

                110       120       130       140       150       160       170       180       190       200
EHE12    RQFSYYQGRRIVKAMKTFNTQTCIRFVPRSRQRDYISIKSRGGCYSYLGRTGGKQVVSLAKYGCVYHGIIQHELSHALGFYHEHTRSDRNKYVKINWENV
SseHCEa  S..TSWEKQK.DS...VYHSS.......QNEY.....EN.A..F.A...V..R..L..NRQ..L...VVL..IN.....Q..Q.....DN.......YI
SseHCEb  S..TSWEKQK.DS...VYHSS.......QNEY.....EN.A..F.A...V..R..L..NRQ.......VVL..IN.....Q..Q.....DN.......YI
SseLCE   EKYD.SEKTL.LA..RG.QSK..V..I..AT..A.L..EP.Y..S.L..Y..D...L..QR...INN......ML..............DQ.IR.....I
SseHE    DYYYDSEKAS.L...E..HQK..V....HRG.T..L..E.EL..W.SV..D..H.....SV...LE..T.......L.......H.........DR..R...G..

                210       220       230       240       250       260       270
EHE12    APRSIYNFQKQNTNNLNTPYDYTSIMHYGKTAFSTN-GKDTITPIPNPKQSIGQRRSMSKGDILRIKKLYNCCKKRNI.
SseHCEa  N.QNA...Y...............V....R...TVQY.R.........NVQ....PG..RW..I..NA..G..
SseHCEb  N.QNA...Y.............S.V....R...TVQY.R.........NVQ.....G..RW..I..NA..G..
SseLCE   NKYFV...R.MD.D.........S.V....R...G.Q-RQE....TYD.SAT....EGL.NI..F..N...R..-WGFSG.
SseHE    QAMNK.D.H.KD............V....R.V..SGF.-.......DSSVAV..SKE..DI.....NR..R.----...
```

B)

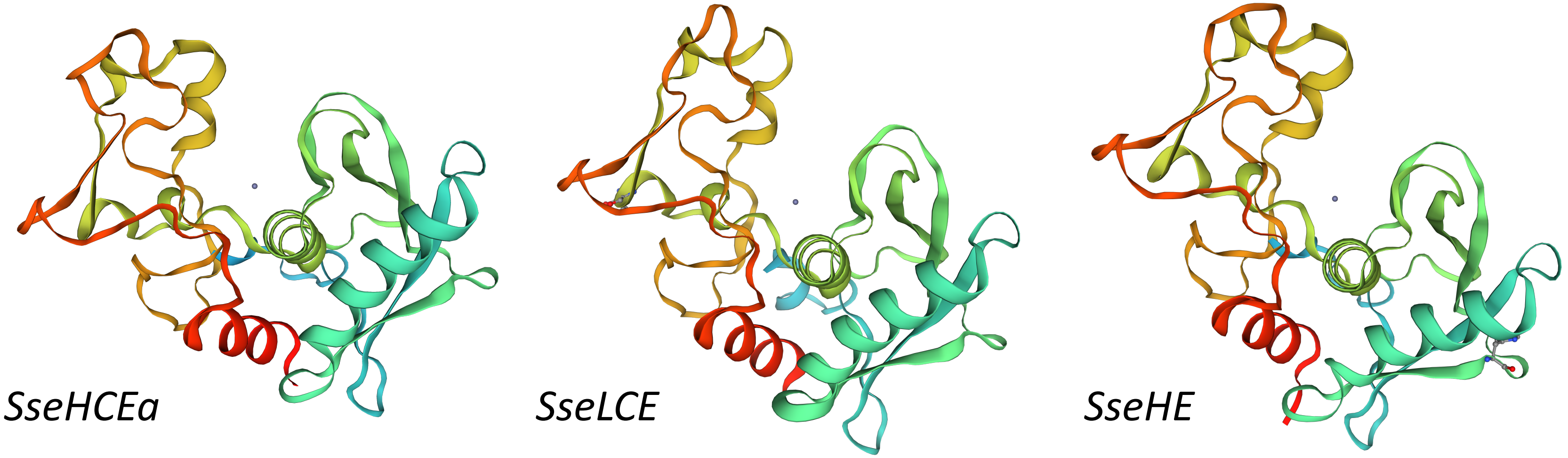

Supplement: S1 Fig — A) Multiple alignment of amino acid sequences of EHE12 (Anguilla japonica) and SseHCEa, SseHCEb, SseLCE and SseHE choriolysins. The signal peptide and propeptide sequences are boxed in light and dark grey, respectively. Dashes and dots represent gaps and identity with respect to EHE12, respectively. Conserved cysteines are indicated in green and the zinc binding motif in light orange. The Met-turn sequence is underlined. B) Three-dimensional structure of SseHCEa, SseLCE and SseHE proteins. (PDF) [file pone.0225666.s003.pdf]
