# Supplementary material for: Genomic and phylogenetic analysis of choriolysins, and biological activity of hatching liquid in the flatfish Senegalese sole
Source: PLoS One. 2019 Dec 5;14(12):e0225666. doi: 10.1371/journal.pone.0225666 (PMC6894847; doi:10.1371/journal.pone.0225666)
Supplement: S2 Fig — The lateral, ventral and dorsal views are shown for antisense probes. (PDF) [file pone.0225666.s004.pdf]

SseHCEa

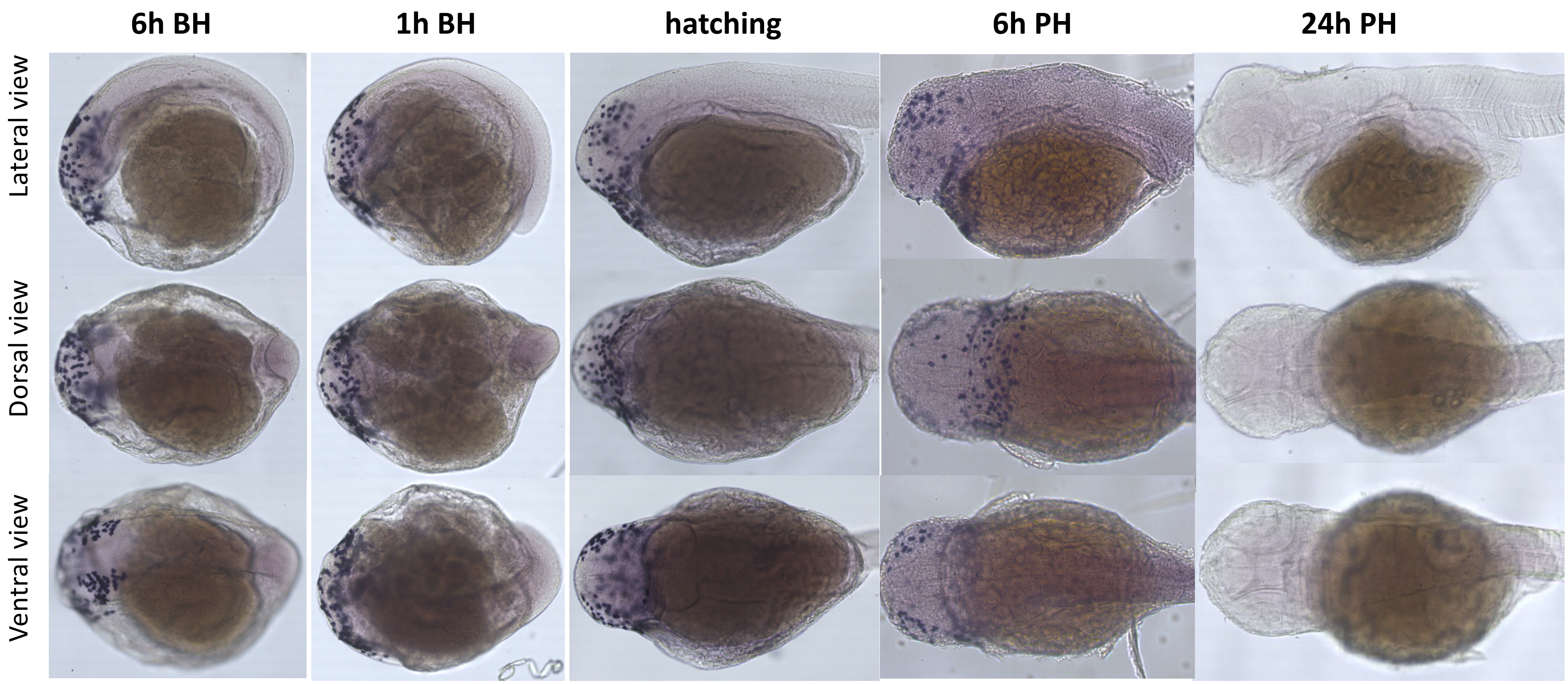
6h BH
1h BH
hatching
6h PH
24h PH
Lateral view
Dorsal view
Ventral view

SseHCEb

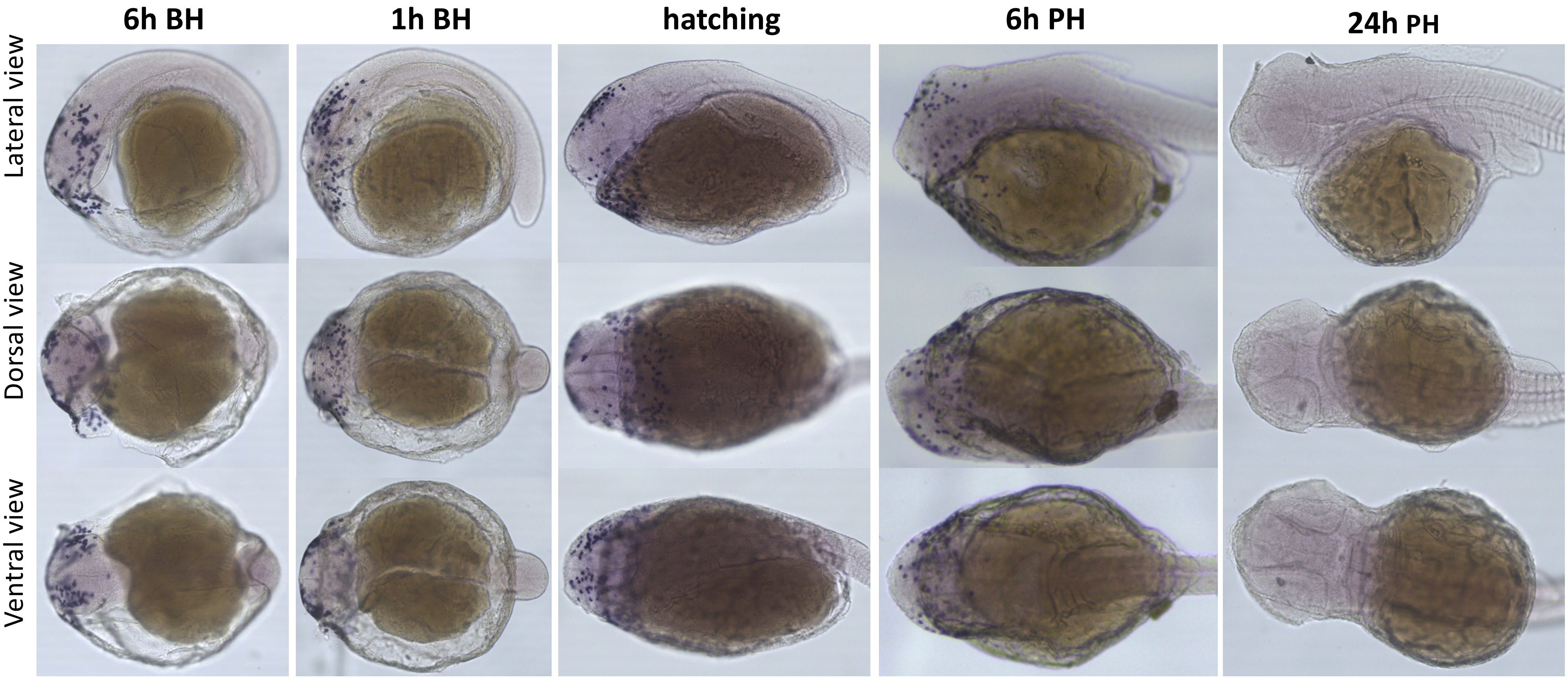
6h BH
1h BH
hatching
6h PH
24h PH
Lateral view
Dorsal view
Ventral view

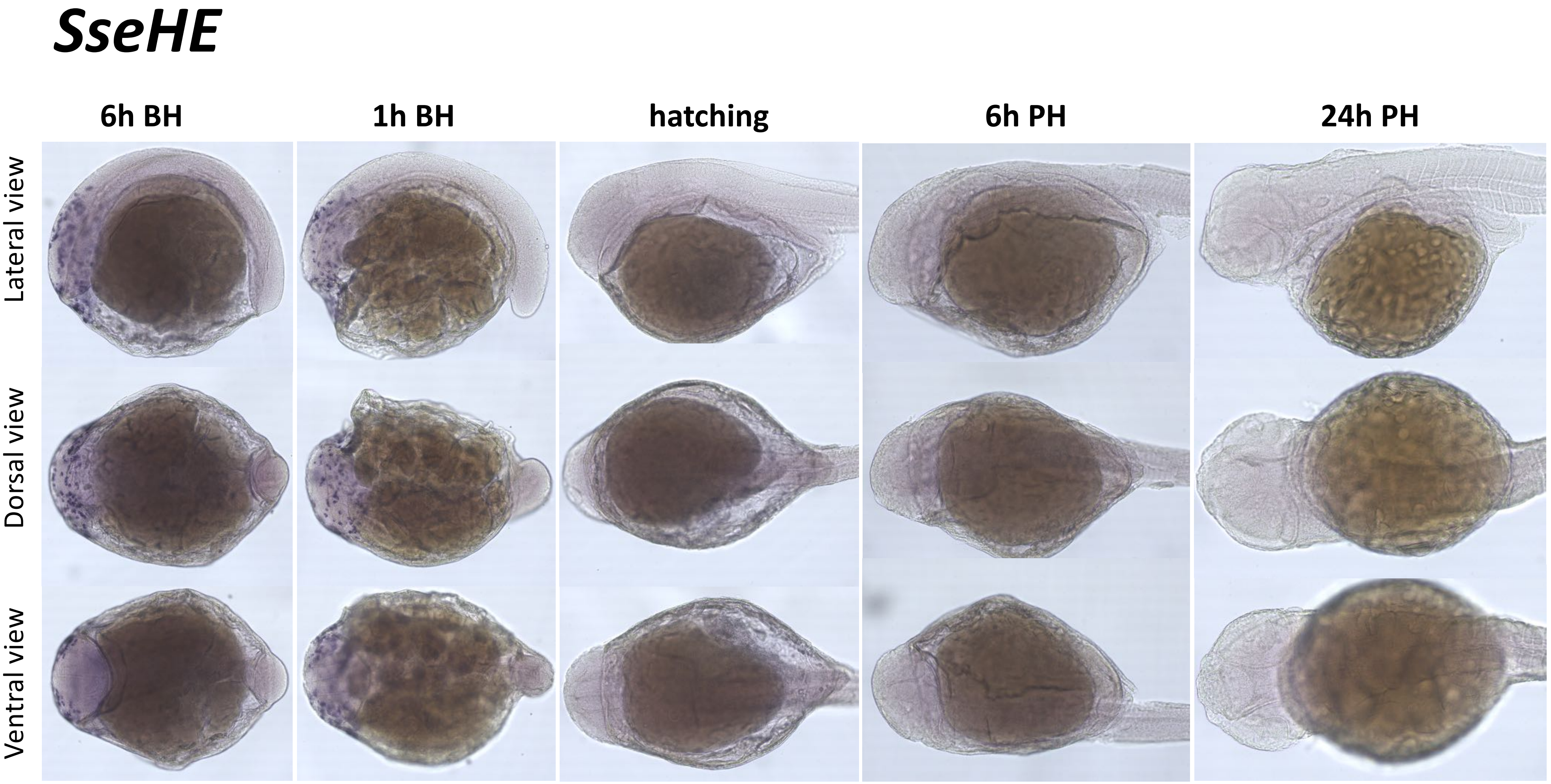
SseHE
6h BH
1h BH
hatching
6h PH
24h PH
Lateral view
Dorsal view
Ventral view

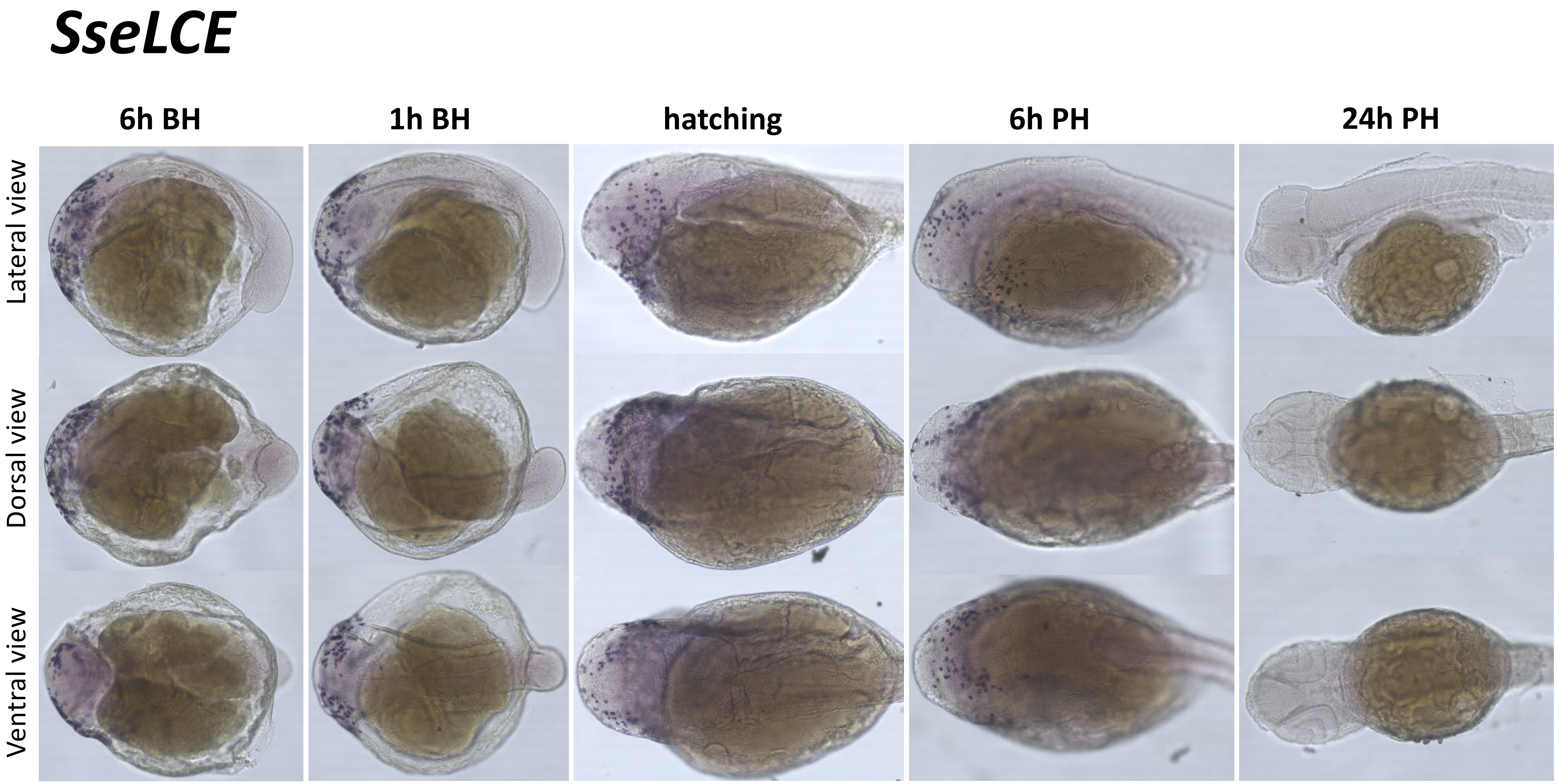

SseLCE
6h BH
1h BH
hatching
6h PH
24h PH
Lateral view
Dorsal view
Ventral view

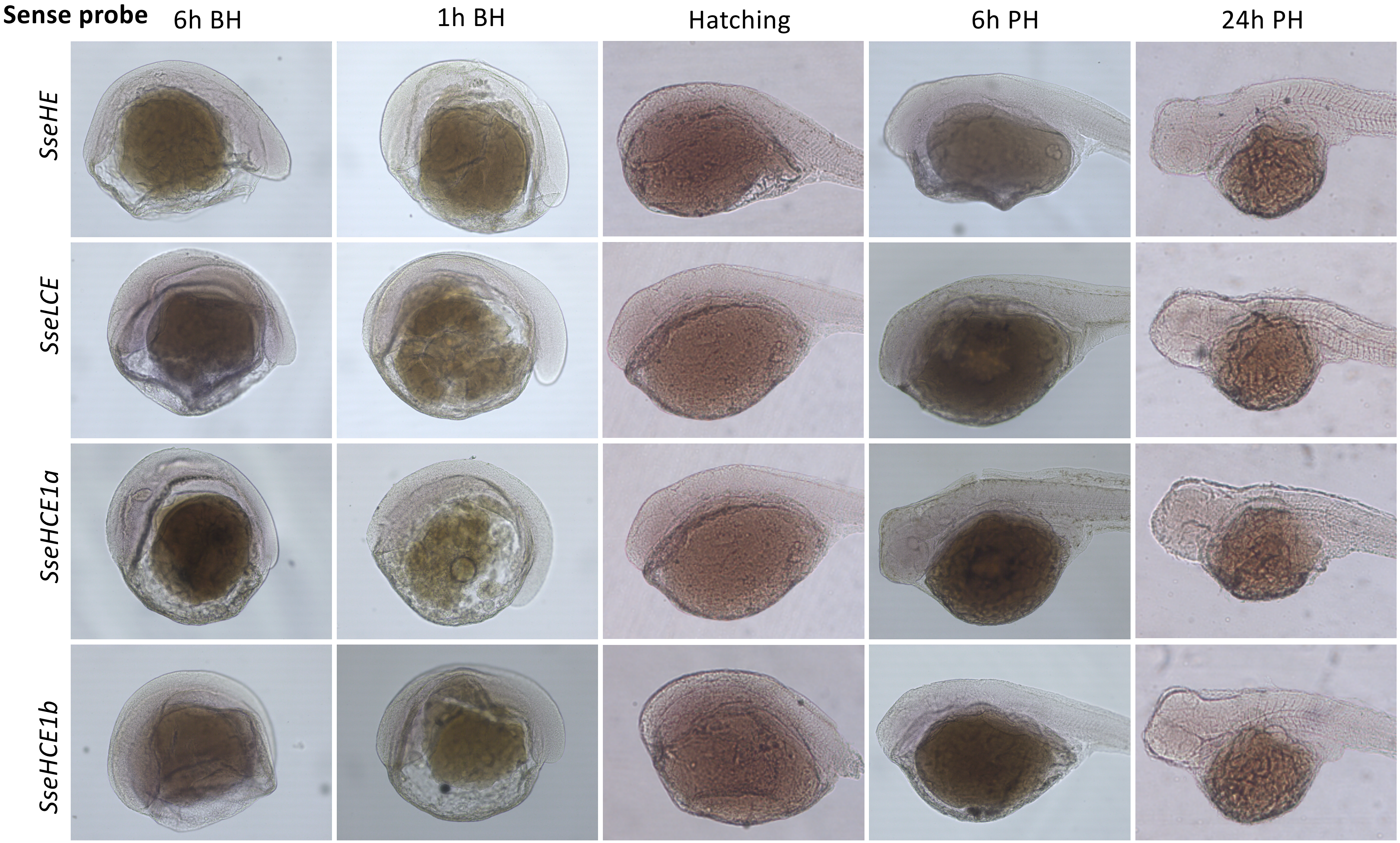
Sense probe
6h BH
1h BH
Hatching
6h PH
24h PH
SseHE
SseLCE
SseHCE1a
SseHCE1b
