# Supplementary figures and images for: Genomic and phylogenetic analysis of choriolysins, and biological activity of hatching liquid in the flatfish Senegalese sole
Source: PLoS One. 2019 Dec 5;14(12):e0225666. doi: 10.1371/journal.pone.0225666 (PMC6894847; doi:10.1371/journal.pone.0225666)

180 -  
130 -  
100 -  
75 -  
63 -  
48 -  
35 -  
28 -  
17 -

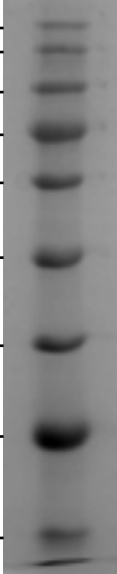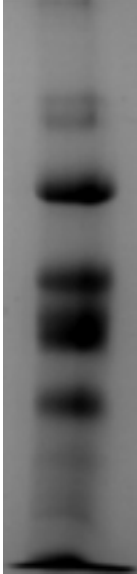

Supplement: S3 Fig — The figures on the left refer to the molecular weights (kDa) of the markers. (PDF) [file pone.0225666.s005.pdf]
