# Supplementary material for: Genomic and phylogenetic analysis of choriolysins, and biological activity of hatching liquid in the flatfish Senegalese sole
Source: PLoS One. 2019 Dec 5;14(12):e0225666. doi: 10.1371/journal.pone.0225666 (PMC6894847; doi:10.1371/journal.pone.0225666)
Supplement: S1 raw images — Loading order, sample identifiers, method used to capture the image, and the the selected lanes from that original image are indicated. (PDF) [file pone.0225666.s006.pdf]

SDS polyacrylamide gel electrophoresis of hatching liquid

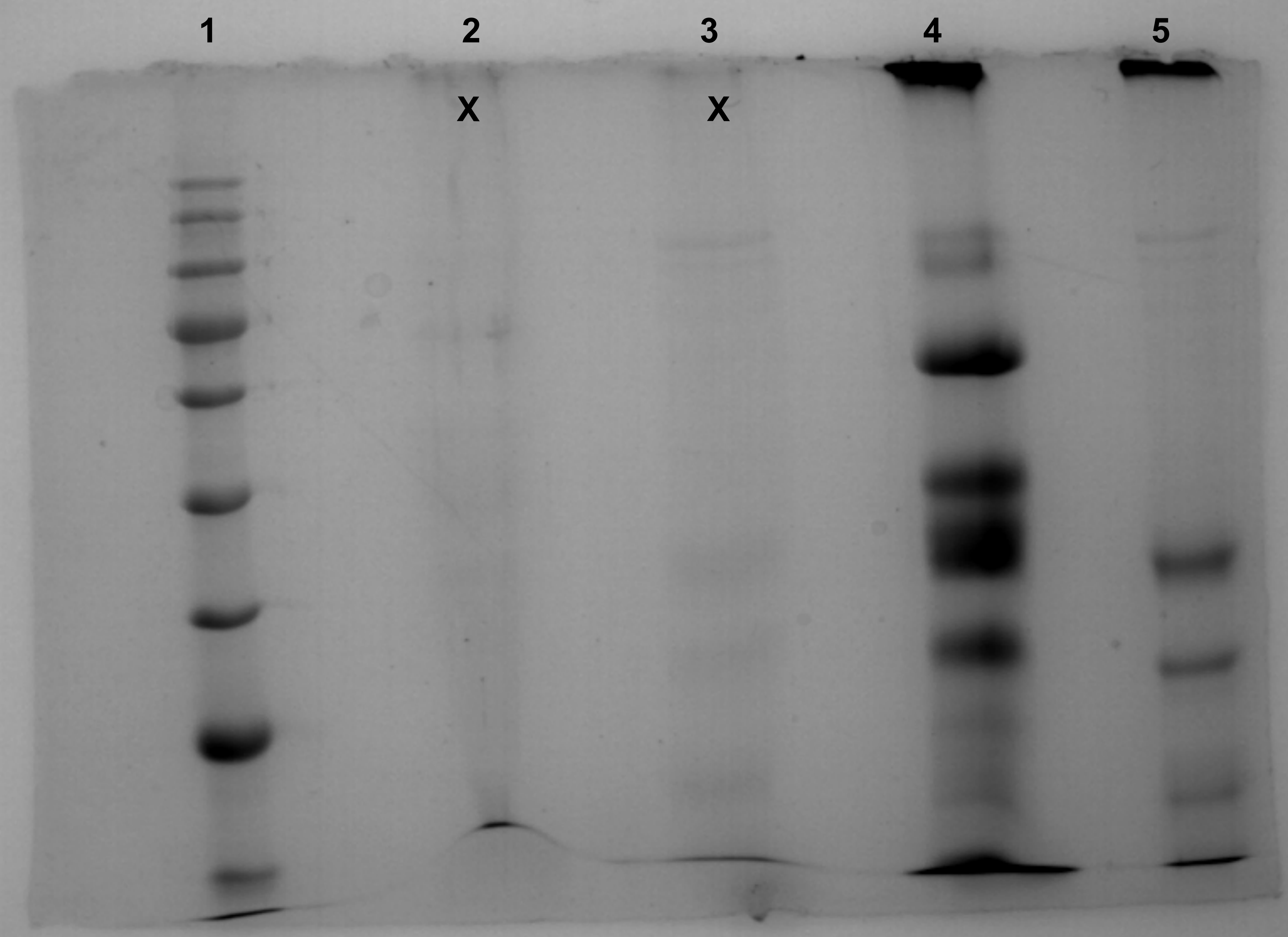

The image was taken using MiniBIS Pro DNR Bioimaging systems

Lane 1: ladder BlueStar Prestained Protein Marker (Nippon) range: 10 – 180 kDa

Lanes 2 and 3 are non-dialyzed samples of two hatching liquid batches

Lanes 4 and 5 are the same two hatching liquid batches after dialysis: Lane4 is included in S3 Fig and lane 5 in Fig 6A.

Zymography of two hatching liquid batches

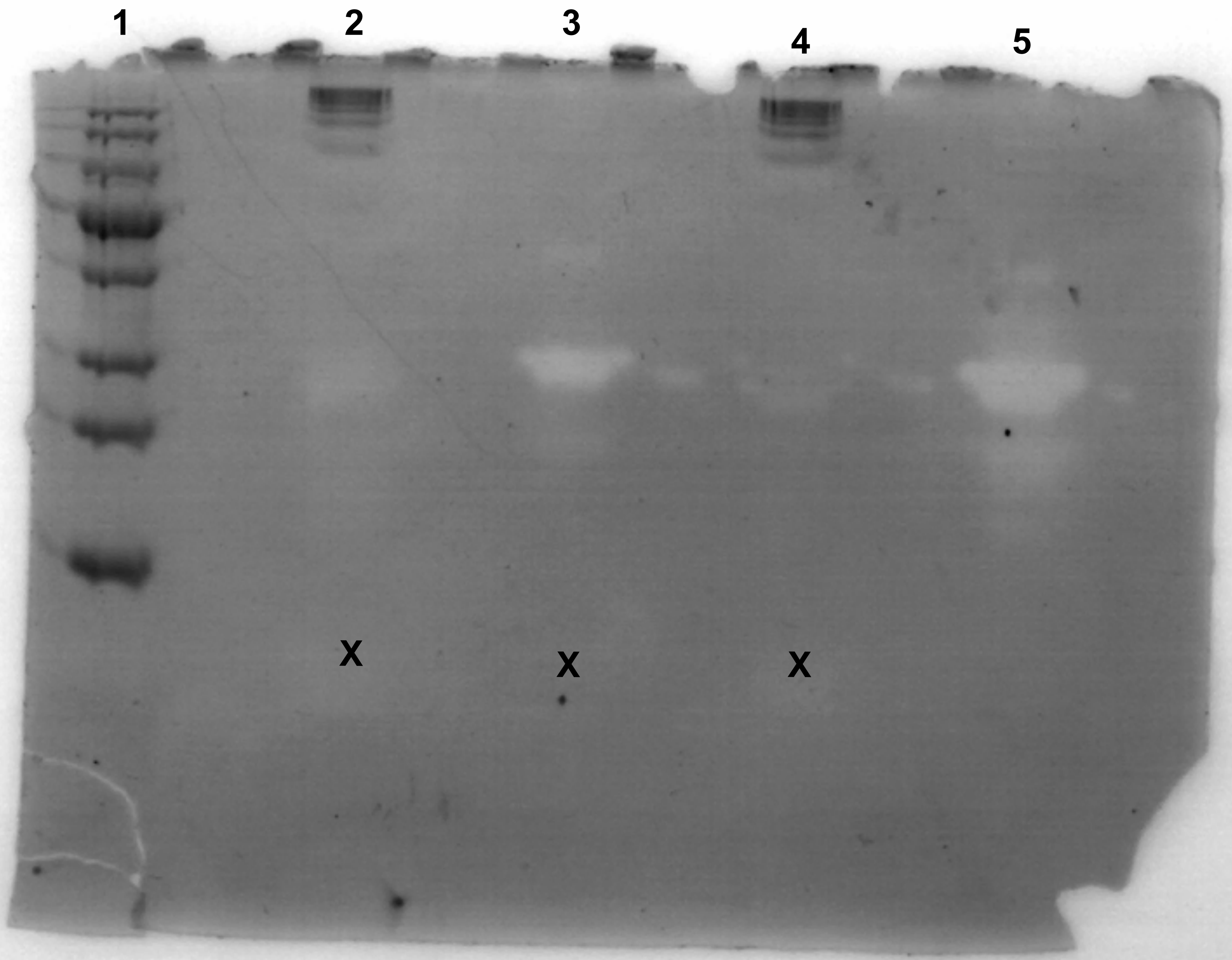

The image was taken using MiniBIS Pro DNR Bioimaging systems

Lane 1: ladder BlueStar Prestained Protein Marker (Nippon) range: 10 – 180 kDa

Lanes 2 and 4 are non-dialyzed samples of two different hatching liquid batches

Lanes 3 and 5 are dialyzed samples of the same hatching liquid batches. Sample 5 is represented in Fig. 6B
